# Supplementary material for: Mapping the evolution of fertility support policies in China: A content and instrumental analysis
Source: PLoS One. 2025 Oct 9;20(10):e0332137. doi: 10.1371/journal.pone.0332137 (PMC12510515; doi:10.1371/journal.pone.0332137)
Supplement: S1 Appendix — (ZIP) [file pone.0332137.s001.zip › S1 Appendix. 226 original policy documents/46-国家卫生健康委员会、国家中医药管理局、国家疾病预防控制局关于印发“十四五”全民健康信息化规划的通知(FBM-CLI.4.5138234).doc]

**国家卫生健康委员会、国家中医药管理局、国家疾病预防控制局关于印发“十四五”全民健康信息化规划的通知**

发布部门： [国家卫生健康委员会](https://www.pkulaw.com/law/chl?Aggs.IssueDepartment=60321&way=textBasic) [国家中医药管理局](https://www.pkulaw.com/law/chl?Aggs.IssueDepartment=7060306&way=textBasic) [国家疾病预防控制局](https://www.pkulaw.com/law/chl?Aggs.IssueDepartment=60434&way=textBasic)

发文字号：国卫规划发〔2022〕30号

发布日期：2022.11.07

实施日期：2022.11.07

时效性： [现行有效](https://www.pkulaw.com/law/chl?Aggs.TimelinessDic=01&way=textBasic)

效力级别： [部门工作文件](https://www.pkulaw.com/law/chl?Aggs.EffectivenessDic=XE0303&way=textBasic)

法规类别： [卫生综合规定](https://www.pkulaw.com/law/chl?Aggs.Category=09601&way=textBasic)

国家卫生健康委员会、国家中医药管理局、国家疾病预防控制局关于印发“十四五”全民健康信息化规划的通知
（国卫规划发〔2022〕30号）

各省、自治区、直辖市及新疆生产建设兵团卫生健康委、中医药局，国家卫生健康委机关各司局、委直属和联系单位、中国老龄协会，国家中医药局、国家疾控局机关各司局、各直属单位：
　　为推动“十四五”期间全民健康信息化发展，国家卫生健康委、国家中医药局、国家疾控局制定了《“十四五”全民健康信息化规划》。现印发给你们，请认真贯彻执行。

国家卫生健康委
　　国家中医药局
　　国家疾控局
　　2022年11月7日

“十四五”全民健康信息化规划

　　“十四五”时期是全民健康信息化建设创新引领卫生健康事业高质量发展的重要机遇期，也是以数字化、网络化、智能化转型推动卫生健康工作实现质量变革、效率变革、动力变革的关键窗口期。为抢抓信息革命机遇，加快全民健康信息化建设，培育行业发展新动能，为实施健康中国战略、积极应对人口老龄化战略、构建优质高效的医疗卫生服务体系提供强力支撑，根据《[中华人民共和国国民经济和社会发展第十四个五年规划和2035年远景目标纲要](https://www.pkulaw.com/chl/dad03bd96074290cbdfb.html?way=textSlc)》《“十四五”国家信息化规划》《“十四五”国民健康规划》和《“十四五”推进国家政务信息化规划》等文件精神，编制本规划。
　　**一、**现状与形势
　　“十三五”期间，卫生健康行业大力推进健康中国、数字中国两大战略融合落地，深入实施“十三五”全民健康信息化发展规划，加快健康医疗大数据规范应用和“互联网＋医疗健康”创新发展，顺利完成各项任务，为支撑卫生健康事业高质量发展发挥了重要作用，取得了显著成效。
　　（一）制度规范的顶层设计基本形成。出台《关于[促进和规范健康医疗大数据应用发展的指导意见](https://www.pkulaw.com/chl/3916c4f95baa8e8bbdfb.html?way=textSlc)》《关于[促进“互联网＋医疗健康”发展的意见](https://www.pkulaw.com/chl/37395b41f6f018e4bdfb.html?way=textSlc)》，制定实施“十三五”全民健康信息化发展规划和安全规划，初步形成以信息化建设为基础、以大数据发展和“互联网＋”服务为引领的“一体两翼”发展格局。印发《关于[加强全民健康信息标准化体系建设的意见](https://www.pkulaw.com/chl/6c5528153b4fe48cbdfb.html?way=textSlc)》，制定实施医院、基层医疗卫生机构和公共卫生信息化建设标准与规范、省统筹区域全民健康信息平台和医院信息平台应用功能指引、医院信息化建设应用技术指引，推进病案首页书写规范、疾病分类与代码、手术操作分类与代码、医学名词术语“四统一”，发布220多项卫生健康信息化标准，逐步实现信息化建设“书同文”“车同轨”。
　　（二）互联互通的平台基础逐步夯实。国家全民健康信息平台初步建成，省统筹区域全民健康信息平台不断完善，实现各级平台联通全覆盖。建立健全全员人口信息、居民电子健康档案、电子病历和基础资源等数据库，强化医疗服务、医疗保障、药品供应等应用系统数据集成和业务协同。积极推动公立医院逐步接入区域全民健康信息平台，依托平台推动不同医疗机构之间诊疗信息互通共享。全国建成1700多家互联网医院，7000多家二级以上公立医院接入区域全民健康信息平台，260多个城市实现区域内医疗机构就诊“一卡（码）通”，2200多家三级医院初步实现院内互通。
　　（三）疫情防控的应急能力全面提升。发挥大数据在疫情防控、监测分析、病毒溯源、物资调配等方面的重要作用，搭建跨部门数据共享平台，强化部门协同、信息联动、数据共享，支撑重点人群排查与密接人员追踪，降低社会风险。“互联网＋医疗健康”发挥突破时空限制免接触优势，在保障患者就医需求、降低患者感染风险等方面发挥了重要作用，开辟了线上抗疫战场。运用大数据追踪风险人群，提高排查工作精准性、及时性，支撑做到“四早”“四清”。推进健康码政策统一和标准一致，实现核酸检测结果和新冠病毒疫苗接种信息全国共享，保障群众有序出行，高效统筹疫情防控和社会经济发展。
　　（四）便民服务的应用成效不断凸显。推进业务协同体系建设，全国二级及以上医院全面推进落实“互联网＋医疗健康”10项服务30条措施，深化便民惠民“五个一”服务行动，全国各级医院普遍开展互联网健康咨询、分时段预约就诊、诊间结算、医保联网、检查检验结果查询、移动支付等线上服务，优化改造就医流程，看病就医“三长一短”问题得到有效缓解。全国远程医疗协作网覆盖地级市和所有国家级贫困县，实现优质医疗资源下沉基层特别是偏远农村地区，有力促进“重心下移、资源下沉”。推动政务服务事项跨地区远程办理、跨层级联动办理、跨部门协同办理，构建便民服务“一张网”，“互联网＋政务服务”效能大幅提升。
　　（五）网络安全的防护能力明显增强。贯彻《[网络安全法](https://www.pkulaw.com/chl/4dce14765f4265f1bdfb.html?way=textSlc)》等相关法律法规要求，印发《国家健康医疗大数据标准、安全和服务管理办法（试行）》，制定卫生健康行业关键信息基础设施认定规则。建立卫生健康行业网络信息与数据安全责任制。健全网络安全治理体系，制定网络安全事件应急预案，完成重大活动期间网络安全保障任务，全面提升网络安全防护能力。加大网络安全管理和技术培训力度，组建网络安全专家队伍和技术支撑队伍，举办卫生健康行业网络安全技能大赛，开展全行业网络安全监测，不断提高快速处置网络安全事件能力，切实提升网络安全保障水平。
　　总体来看，“十三五”期间我国全民健康信息化建设成效显著，但目前仍处在夯台垒基、爬坡过坎的关键时期，在基础设施、共享应用、投入保障、网络安全等方面还存在短板与弱项，特别是统筹协调机制还不健全，法规标准建设有待强化，信息化建设投入机制有待完善，专业人才较为匮乏，数据要素价值潜力尚未充分激活，“数字鸿沟”“数据壁垒”依然存在，网络安全形势严峻复杂，数据治理能力有待进一步提升。从国际上看，全球加速迈进数字化发展快车道，特别是新冠肺炎疫情深刻冲击和挑战全球医疗卫生体系，数字技术在卫生健康领域的应用更加广泛、影响更加深刻。面对数字化变革带来的机遇与挑战，必须进一步夯实全民健康信息化新基建，培育卫生健康服务新业态，提升卫生健康行业发展新动能，构建数据要素治理新格局，努力实现全民健康信息化建设更高质量、更有效率、更加公平、更可持续、更为安全的发展新局面。

　　**二、**总体思路
　　（一）指导思想
　　以习近平新时代中国特色社会主义思想为指导，深入贯彻落实党的二十大精神，紧密结合卫生健康行业应用需求和新一代信息技术发展大势，把握问题导向、需求导向和应用导向，统筹发展和安全，强化系统思维，以引领支撑卫生健康事业高质量发展为主题，促进全民健康信息服务体系化、集约化、精细化发展，进一步畅通全民健康信息“大动脉”，以数据资源为关键要素，以新一代信息技术为有力支撑，以数字化、网络化、智能化促进行业转型升级，重塑管理服务模式，实现政府决策科学化、社会治理精准化、公共服务高效化，为防范化解重大疫情和突发公共卫生风险、建设健康中国、推动卫生健康事业高质量发展提供坚强的技术支撑。
　　（二）基本原则
　　坚持统筹集约，共建共享。坚持统筹布局，深化共建共用，增强全民健康信息化发展的系统性、整体性和协调性，以构建大平台、大系统、大目录为导向，加大信息化建设统筹力度，加强信息化基础设施集约化建设，巩固政务信息系统整合成果，进一步破除数据共享壁垒，畅通数据共享通道，推进数据全生命周期管理。
　　坚持服务导向，业务驱动。坚持以人民为中心的发展思想，以信息赋能为关键，以优质服务为导向，以智慧决策为基础，以协同治理为手段，形成应用牵引建设、服务促进联通的发展机制，推进信息与业务深度融合，进一步降低服务成本，缩小“数字鸿沟”，发展和推广便民惠民服务，推动工作重心下移、优质资源下沉，提升卫生健康服务均等化、普惠化、便捷化水平。
　　坚持开放融合，创新发展。充分发挥新一代信息技术的优势，构建基于数据驱动的生态系统，强化区域数据汇聚应用，推进跨部门、跨地域、跨层级、跨系统、跨业务的技术融合、数据融合、业务融合，创新数据供给方式，深化数据开发利用，促进行业转型升级，推动关键技术和服务模式创新，推进健康医疗数据资源和基础设施开放共享，不断提高卫生健康行业治理水平。
　　坚持规范有序，安全可控。树立科学的网络安全观，坚持发展与安全并重，把安全治理贯穿全民健康信息化建设管理应用全过程，划定监管底线和红线，构建权责可界定、过程可追溯、安全可审计的制度规则，切实防范化解风险，建立健全平台经济治理体系，规范资本参与和监管，促进公平和有序竞争，确保数据安全和网络安全。
　　（三）发展目标
　　到2025年，初步建设形成统一权威、互联互通的全民健康信息平台支撑保障体系，基本实现公立医疗卫生机构与全民健康信息平台联通全覆盖。加速推进高速泛在、云网融合、智能敏捷、集约共享、安全可控的全民健康信息化基础设施建设。依托国家电子政务外网、互联网、光纤宽带、虚拟专线和5G等网络建设完善卫生健康行业网。全民健康信息化统筹管理能力明显增强，全国医疗卫生机构互通共享取得标志性进展，二级以上医院基本实现院内医疗服务信息互通共享，三级医院实现核心信息全国互通共享。全员人口信息、居民电子健康档案、电子病历和基础资源等数据库更加完善。数字健康服务成为医疗卫生服务体系的重要组成部分，每个居民拥有一份动态管理的电子健康档案和一个功能完备的电子健康码，推动每个家庭实现家庭医生签约服务，建成若干区域健康医疗大数据中心与“互联网＋医疗健康”示范省，基本形成卫生健康行业机构数字化、资源网络化、服务智能化、监管一体化的全民健康信息服务体系。

　　**三、主要任务
　　（一）集约建设信息化基础设施支撑体系
　　统筹推动全民健康信息平台建设，鼓励地方结合实际，探索多种方式，采取“国家和省两级部署，国家、省、市、县四级应用”总体框架，集约建设各级全民健康信息平台和传染病监测预警与应急指挥信息平台，全面推进医疗卫生机构信息化建设提档升级，鼓励各地因地制宜构建全民健康基础设施云，推动数字健康新型基础设施建设，全方位提升卫生健康信息化基础设施水平。
　　完善国家全民健康信息平台功能。完善国家卫生健康委政务云基础设施建设和国家全民健康信息平台功能，依托国家电子政务外网和互联网等网络，推动互联互通和数据共享，提升国家与省统筹区域全民健康信息平台的信息枢纽能力。建立国家级个人健康信息索引，支撑实现跨省电子病历、居民电子健康档案查询。完善全民健康信息平台应用支撑、服务注册、资源目录、门户管理等能力，开展应用评价，完善平台“建管用”评价促进机制。加强系统和资源整合，强化数据分析应用，推进数据可视化，实现数据统一标准、一次采集、整合共享、多方利用。
　　加强省统筹区域全民健康信息平台建设。以建立统一的云基础设施为支撑，构建省统筹区域全民健康信息平台，支撑省、市、县三级应用，推进一体化的数据采集、汇聚、治理、共享和分析应用管理。因地制宜以实体或虚拟方式建立市级、县级全民健康信息平台。以平台为载体整合业务系统，构建功能一致、融合开放、有机对接、授权分管的平台基础功能，逐步实现所有医疗卫生机构规范接入各级全民健康信息平台，纵向联通上下级全民健康信息平台，横向联通同级政府相关部门信息平台，畅通部门、区域、行业之间的数据交换。探索推动社会化健康医疗大数据向各级信息平台集聚。
　　构建传染病监测预警与应急指挥信息平台。以全民健康信息平台为基础，按照“整体统筹、横向整合、纵向贯通、重点突出”的原则，建立以疾控机构为支撑，以国家级传染病监测预警与应急指挥信息平台为主体，各省省统筹区域传染病监测预警与应急指挥信息平台共建、共治、共享，实现国家与省统筹区域两级平台，覆盖国家、省、市、县四级应用。融合多源数据，拓展异常健康事件、严重症候群、病原学检测、媒体舆情、社会举报等报告渠道，实现对传染病疫情和突发公共卫生事件的快速响应和高效调度处置，为防范化解重大疫情和突发公共卫生事件提供有力支撑。
　　全面推进医院信息化建设提档升级。将信息化作为医院基本建设的优先领域。按照《全国医院信息化建设标准与规范》《全国公共卫生信息化建设标准与规范》要求，二级及以上医院持续完善医院信息平台功能，整合医院内部信息系统。推进医院新一代数据中心建设，实现医疗业务协同一体化、惠民医疗服务一站式、精准决策支持一门户、信息资源管理一张图、数据分析利用一平台、数据资源汇聚一个库，强化对医院精细化运行管理和全视角决策评价的技术支撑和数据保障。鼓励医院信息系统云上部署，推进医学影像数据存储、互联网服务和应用信息系统分步上云。专科医院参照相应等级综合医院要求，根据医院专科特色和发展需求开展信息化建设。**

专栏一 全民健康信息新基建强化工程

1.加强全民健康信息平台基础设施建设。落实《“十四五”推进国家政务信息化规划》，拓展国家卫生健康委政务云基础设施，增强信息系统快速部署和弹性扩展能力。

2.提高全民健康信息平台业务支撑能力。发挥平台作为卫生健康政务服务的枢纽作用，支撑“互联网＋医疗健康”服务，优化“互联网＋政务服务”，完善“互联网＋监管”。支撑医联体、医疗联盟、国家区域医疗中心等跨机构跨层级跨地域的业务应用，加强部门间信息共享。推动IPv6、北斗等在卫生健康领域的应用。

3.提高全民健康信息平台决策支撑能力。发挥平台“健康大脑”作用，集成卫生资源、医疗服务、公共卫生、健康状况、医改监测、食品安全与营养健康等专题指标数据，通过数据的横向对比与纵向查询，提供简洁、方便的操作，加强数据可视化应用，强化决策支撑。

4.强化数据深度挖掘与分析应用。完善全员人口信息、居民电子健康档案、电子病历和基础资源等数据库。加强基础资源数据库建设，逐步实现医疗机构、医护人员、应急救治、医疗设备、药品耗材、健康管理、产业发展和信息服务等健康医疗基础数据和公共信息资源集聚整合，实现对数据深度挖掘。

5.加快建立全国“一盘棋”的监测预警和应急处置协同新模式。以国家级传染病监测预警与应急指挥信息平台为主体，以推进全国传染病监测预警与应急指挥信息体系建设为目标，建设完善传染病监测预警与应急指挥信息平台，增强传染病疫情和突发公共卫生事件早期监测预警能力，提高疫情防控和突发公共卫生事件应急处置水平。

6.建设集医学科学数据、医学研究登记备案信息、研究成果等为一体的国家级医学研究科技资源基础信息平台及综合服务平台，推动国家医学研究登记备案信息系统与生物医学文献服务系统、生物样本库、动物模型资源库等基础研究平台实现对接。加强毕业后医学教育信息管理系统建设。

　　（二）健全全民健康信息化标准体系
　　落实《[标准化法](https://www.pkulaw.com/chl/36bdf552f9bcf06cbdfb.html?way=textSlc)》，坚持“统筹规划、急用先行、规范有序、协同高效”的原则，逐步形成统一权威、全面协调、管理规范、自主可控的全民健康信息化标准体系。
　　完善全民健康信息化应用基础标准。按照《关于[加强全民健康信息标准化体系建设的意见](https://www.pkulaw.com/chl/6c5528153b4fe48cbdfb.html?way=textSlc)》要求，研究制订唯一对象标识、对象注册与解析、临床医学术语、检查检验代码、药品耗材应用编码、数据交互接口、数据分析、数据质量、临床决策支持等基础标准，加快健全完善网络安全等级保护、数据安全、个人信息保护等标准体系。推动完善健康医疗大数据、“互联网＋医疗健康”、医学人工智能及5G、区块链、物联网等新一代信息技术标准体系和统一规范的国家中医药数据标准和资源目录体系，支撑在应急救治、远程会诊、远程检查、临床辅助诊断决策、公共卫生服务、医院管理等方面应用。鼓励医疗卫生机构、科研院所、高等院校、学会协会、企业等参与团体标准和地方标准的研制工作。积极参与国际标准化组织工作，参与国际标准制订，提升标准国际影响力。
　　加强全民健康信息化标准应用推广。全面推进基础类、数据类、应用类、技术类、管理类、安全与隐私类等6大类全民健康信息化基础标准在卫生健康行业落地实施，推进病案首页书写规范、疾病分类与代码、手术操作分类与代码、医学名词术语“四统一”。落实全国统一的医疗机构、医护人员等基础资源及信息互联互通编码标准。加强省级区域居民电子健康档案和电子病历数据标准统筹，统一区域全民健康信息平台与医院信息平台的数据接口标准。整合医疗机构内部信息系统，使用统一的数据接口实现共享交换。加强医院、基层医疗卫生机构、公共卫生机构信息化标准建设。
　　深化全民健康信息化标准服务管理。建立健全国家全民健康信息化标准服务平台，完善卫生健康信息标准元数据管理功能，为标准的研制使用提供技术支撑。强化标准应用程度和建设成效评价，统筹规范有序开展标准应用情况测评，分类分层推进各级各类医疗卫生机构标准化评价，持续推动医疗健康信息互联互通标准化成熟度测评、电子病历系统应用水平分级评价和医疗卫生机构信息化标准建设“自评价”，稳步推进信息化标准评价一体化。加强标准应用成果总结宣传，推广各地标准化建设应用的创新典型案例，提升社会各方的标准化意识和自主标准使用能力。
　　（三）深化“互联网＋医疗健康”服务体系
　　总结“互联网＋医疗健康”支撑新冠肺炎疫情防控经验，将其制度化、常态化，完善“互联网＋医疗健康”服务体系，进一步拓展“互联网＋医疗健康”服务模式，优化资源配置，提高服务效率，降低服务成本，满足人民群众日益增长的卫生健康需求。
　　拓展“互联网＋医疗健康”服务。进一步贯彻落实国务院办公厅《关于[促进“互联网＋医疗健康”发展的意见](https://www.pkulaw.com/chl/37395b41f6f018e4bdfb.html?way=textSlc)》，健全“互联网＋医疗健康”服务体系。持续开展“互联网＋医疗健康”“五个一”服务行动，推进10项服务30条措施落地落实，构建线上线下深度融合覆盖全生命周期的卫生健康服务模式。大力发展远程医疗，推动优质医疗资源扩容下沉和均衡布局，提高卫生健康服务均等化与可及性。推进“互联网＋家庭医生签约服务”“互联网＋妇幼健康”“互联网＋医养服务”“互联网＋托育服务”“互联网＋营养健康”等，提高重点人群健康服务智能化、专业化水平。开展“互联网＋护理服务”，强化与家庭医生签约、延续性护理等服务有机结合，为群众提供个性化、差异化的护理服务。开展“互联网＋心理健康服务”，探索构建覆盖全人群、服务全生命周期、提供全流程管理的心理健康和精神卫生服务管理体系。探索开展“互联网＋药学服务”模式，推广电子处方区域流转。
　　加强“互联网＋政务服务”。依托各级“互联网＋政务服务”平台，强化身份认证、电子印章、数据共享等基础支撑，优化政务服务流程，推进线上线下深度融合，实现卫生健康政务服务事项应上尽上。持续深化生育登记、义诊活动备案、医疗广告审查、消毒产品卫生安全评价报告备案等政务服务跨省通办，实现企业和群众异地办事“马上办、网上办、就近办、一地办”。深入推进出生医学证明电子证照跨地区、跨部门共享，做好出生医学证明电子证照文件在线核验、共享复用工作，全面推广“出生一件事”，方便群众办事，提升政务服务效能。依托全国一体化政务服务平台，拓展电子证照应用领域和证照免提交范围，推动全国互通互认。依托国家级和省统筹区域全民健康信息平台，做好人口死亡登记数据等网络直报信息向基层回流，实现数据一次采集、多方利用。
　　规范服务保障与监管体系。构建以“双随机、一公开”监管和“互联网＋监管”为基本手段，重点监管为补充，信用监管和在线监管为基础的新型监管机制。依托全民健康信息平台，全面汇聚卫生健康监督、食品安全监管等多层级、多领域、多渠道、多形态的监管数据和关联数据，实现行政审批、行政处罚、监测评估信息互联互通和实时共享。开展线上线下一体化医疗行为监管，确保医疗质量和医疗安全。加强对互联网平台和企业数据行为的监管，运用大数据、人工智能等新一代信息技术实施风险分析和识别，完善个人信息保护，防止数据垄断和商业滥用。

专栏二 数字化智能化升级改造工程

1.依托国家医学中心，统筹建设一批互联互通的重大疾病数据中心，建立主要疾病数据库和大数据分析系统，推进跨地区、跨机构信息系统互通共享。

2.将信息化纳入国家区域医疗中心建设范围，建立远程医疗和教育平台，加快诊疗设备智能化升级改造。支持省级区域医疗中心加强智慧医院建设，保障远程医疗需要，优化服务流程，改善就医体验。

3.加强数字医共体建设。加强县域医共体建设，鼓励依托县级医院建设开发共享的影像、心电、病理诊断、医学检验等中心，加强远程医疗和信息化设备配备，与高水平省市级医院对接，与基层医疗卫生机构联通。在开展紧密型县域医共体建设的地区，建立一体化管理运行和协同服务的信息系统，建设统一的数据库和数据交换中心，提升医共体数字化管理服务能力，实现医共体医疗、预防保健、康复、公共卫生等业务融合集成应用，在医共体内实现就诊一码通行、服务接续、一站式结算，打造数字化智能型医共体。重点支持脱贫地区、三区三州、中央苏区、易地扶贫搬迁安置地区的县级医院完善信息化基础设施配置。

　　（四）完善健康医疗大数据资源要素体系
　　加强健康医疗大数据创新应用和行业治理，以促进数据合规开放共享应用为主线，以提升群众获得感、提高行业治理能力、培育数字经济发展新动能为目标，丰富数据供给，提高数据质量，积极构建健康医疗大数据资源要素体系，推进健康医疗大数据应用发展，充分释放数据价值。
　　加强健康医疗大数据创新应用与行业治理。进一步促进和规范健康医疗大数据应用发展，不断深化在行业治理、临床科研、公共卫生、智能医疗设备等领域的创新应用，积极拓展在疫情防控、监测分析、病毒溯源、物资调配等方面的应用。采取“原始数据不出域、数据可用不可见”等方式，有序推动健康医疗大数据共享应用。建立健全健康医疗数据管理制度，培育健康医疗数据要素市场，激发数据要素价值，推动健康医疗大数据在疾病预防、健康管理、辅助决策、药物研发、医疗保险、精准医疗、营养健康等方面产业化、规模化应用。探索建立数据价值评估体系，完善数据价值评估框架，探索建立政府、高等院校、科研院所、企业等多元协同的健康医疗大数据共治共享机制。
　　强化数据全流程质控和数据治理。强化医疗卫生机构数据源头质控能力，建立覆盖业务全链条的数据采集、传输和汇聚体系，畅通数据汇聚渠道，推进数据模型设计、数据应用技术、数据质量全流程管理等能力建设，提高数据质量。加快推动卫生健康领域公共服务资源数字化供给和网络化服务，促进优质资源共享复用。依托国家健康医疗大数据创新应用示范中心，建设健康医疗大数据资源目录体系，制订健康医疗大数据分类、分级、分域应用规范，形成一批健康医疗公开数据集，推动积极稳妥、安全有序共享开放。
　　推进健康医疗大数据中心建设。总结推广国家健康医疗大数据中心试点建设经验，依托京津冀、长三角、成渝地区双城经济圈、黄河流域等重点区域，加强区域优化布局、集约建设和节能增效，加快构建全国一体化的健康医疗大数据中心协同创新体系。依托省级健康医疗大数据中心，开展区域数据中心和国家健康医疗大数据研究院建设，为健康医疗大数据应用发展提供有力支撑。推动国家健康医疗大数据中心建设，逐步完成健康医疗大数据全国总体战略布局。及时总结、推广、组织一批试点成效好、带动效应强的健康医疗大数据示范项目，加强标杆引领示范作用，形成以点带面、点面结合的良好生态。
　　（五）推进数字健康融合创新发展体系
　　加快数字健康发展和新型基础设施建设，规范促进新一代信息技术在卫生健康领域深度应用，进一步优化要素配置和服务供给，补齐发展短板，提升服务效率，推动健康产业转型升级。
　　构建数字健康战略发展新格局。加强顶层设计和机制建设，推进从生产要素到创新体系，从业态结构到组织形态，从发展理念到服务模式的全方位变革突破，进一步适应数字健康发展新趋势，对接国际标准体系，更好服务和融入新发展格局。推进基础设施、法规标准、数据资源、产业发展、安全保障一体化部署，加强在前沿技术研发、数据开放共享、专业人才培养、隐私安全保护等方面前瞻性布局，健全数字健康的政策法规、伦理标准、人才队伍、数据安全等支撑体系。坚持政府主导、多方参与、联合创新、共建共享，鼓励医疗卫生机构、科研院所、企业等协同创新，加强产业链上下游资源的组织协调，共同营造数字健康良好发展生态。
　　重塑数字健康管理服务新模式。拓展丰富数字健康应用场景和服务空间，构建线上线下一体化服务新模式，提升公共资源供给效率，提高公共服务效用，优化服务流程，改善就医体验，提高群众看病就医的便捷度。发挥居民电子健康档案的基础性载体作用，以家庭医生签约服务为抓手，为城乡居民提供全方位、全生命周期的数字化健康管理服务。拓展数字健康乡村、智慧健康养老、智慧营养膳食、在线医学教育、智慧中医药等服务，不断满足人民群众多层次、多样化、个性化的健康需求。
　　培育数字健康经济产业新业态。聚焦战略前沿推进重点领域数字健康产业发展，立足重大技术突破和重大发展需求，增强产业链关键环节竞争力，完善重点产业供应链体系，加速产品和服务迭代。发展基于数字技术的健康服务，鼓励发展区域检查检验、在线健康咨询、智能慢病管理等多元化、个性化健康服务，催生一批有特色的数字健康管理服务企业。规范发展第三方机构搭建社会化行业服务平台，完善数字健康产业链、供应链和创新链，打造创新发展的数字健康产业生态。构建数字健康科技创新体系，做大做强卫生健康软件产业，增强高性能医疗器械生产装备、医用材料的自主可控能力和国际竞争力，努力将数字健康产业打造成新的经济增长点。
　　提升数字健康行业治理新水平。深入开展数字健康政策、数据综合治理等领域的研究，构建部门协同、资源优化、防治结合、平战一体的运行机制，以服务管理、效率提升、功能完善为导向，感知社会态势、畅通信息渠道、辅助科学决策，提升治理能力的现代化水平。推进政务管理服务电子化、自动化、无纸化，破除体制障碍，打破信息壁垒，逐步实现电子健康码、医保结算码、金融支付码等多码融合、业务通办，解决人民群众办事难、办事慢、办事繁问题。建立全方位、多层次、立体化监管体系，逐步实现医疗就诊记录、费用清单、电子处方、电子病历、医疗费用结算记录等有效监管，不断提升数字健康服务能力和监管水平。

专栏三 新一代信息技术应用促进工程

1.促进医学人工智能应用试点。开展医学人工智能社会治理实验和国家智能社会治理实验特色基地建设，促进医学影像辅助诊断、数字病理辅助诊断、电生理信号辅助诊断、临床辅助决策支持、医院智能管理、公共卫生服务等应用。

2.开展5G＋医疗健康应用试点。围绕急诊救治、远程诊断、远程手术、远程重症监护、中医诊疗、医院管理、智能疾控、健康管理等重点方向，促进5G在卫生健康行业的重点应用创新。

3.开展“区块链＋卫生健康”应用试点。鼓励应用区块链技术加强身份标识管理、卫生健康数据存证、居民电子健康档案共享查阅、药械流通信息追溯、公共卫生事件数据汇聚分析、专病科研数据共享等内容，丰富区块链的应用场景。

4.推进医疗物联网应用试点。发挥物联网泛在连接、低能耗、智能感知的技术优势，围绕智慧病房、远程会诊、重大疫情防控救治等需求，优化远程医疗通信网络基础设施，重点推进智能个人定位、个人可穿戴健康智能监测、具备医疗诊断级性能的生命体征感知等终端设备应用。

5.开展医疗健康机器人应用试点。推进面向卫生健康行业的服务机器人和特种机器人的研制及应用，主要包括手术、护理、检查、康复、咨询、配送等医疗康复机器人及检验采样、消毒清洁、室内配送、辅助巡诊查房、重症护理辅助操作等卫生防疫机器人。

　　（六）拓展基层信息化保障服务体系
　　坚持以基层为重点，加快补齐基层医疗信息化短板，融通汇聚县域内数据，强化数据分析运用，推动基层卫生健康信息化综合治理能力显著提升。
　　强化基层信息化便民服务。规范居民电子健康档案首页，推进居民电子健康档案信息安全有序向个人开放，经授权开展医疗卫生服务查询和健康咨询，探索向居民提供健康画像，推进居民电子健康档案应用。建立个人健康管理便民惠民服务门户，开设线上线下一体化慢病门诊，提供就诊全流程服务，加强诊后跟踪随访、双向转诊等服务。聚焦重点人群，提供线上随访、复检预约等数字化服务。为基层医疗卫生机构配备智能化设备终端及可穿戴设备，自动采集健康数据信息，减少手工填报和纸质报表，推动基层报表通过信息系统直接抓取自动生成，逐步实现基层数据采集只录一次，提供数字化签约、在线续方等线上健康管理，切实为基层减负。通过手机等移动终端，开展健康教育，提高居民健康素养，加强医患在线交流，密切医患关系。
　　强化基层信息化基础设施建设保障。依托省统筹区域全民健康信息平台，以实体或虚拟方式搭建县域基本医疗卫生健康数据中心，加快基层卫生健康信息化基础设施建设和紧密型县域医共体信息系统提档升级，推动基层数据上云。基于县域基本医疗卫生健康数据中心，以居民电子健康档案为载体，围绕居民服务需求和家庭医生签约服务场景，搭建区域数字家医服务平台。支撑整合式医疗的基层医疗卫生信息系统，加强基层医学智能辅助诊断系统在基层的推广应用，整合基本医疗、基本公共卫生、家庭医生签约、运营管理、报表统计分析及中医馆健康信息平台，逐步实现不同层级之间的数据互通共享。推广智能化健康管理设施，方便边远地区、卫生资源薄弱地区的患者就近自助就诊取药。
　　强化基层综合服务监管体系建设。建立一体化综合服务监管体系，开展分级应用，动态掌握基层医疗卫生机构、人员、服务、运行等基本情况，实现基层医疗行为、医疗质量和经费使用等动态监管及药械全过程追溯管理。实施基层卫生健康发展综合绩效评价，推动机构绩效考核自动化、日常化。开展区域健康数据监测、患者疾病谱与就诊流向分析，提升基层卫生治理能力和科学决策水平。在省级统筹下将国家基层卫生健康综合管理平台与县域基本医疗卫生健康数据中心对接联通，建立网络直报信息系统的共享回流机制。

专栏四 基层信息化能力提升工程

1.促进基层综合服务监管数字化。落实《全国基层医疗卫生机构信息化建设标准与规范（试行）》，推进基层医疗卫生机构信息系统、公共卫生管理系统、人口信息系统等业务协同，实现卫生健康信息一体化管理和医疗卫生机构间数据互通共享。

2.推广智慧家医平台建设。利用区域数字家医服务平台，融合签约服务管理、健康管理、诊疗服务、满意度评价、绩效考核等功能，构建家庭医生和签约居民的数字化联系路径，形成以家庭医生和签约居民为双核心的智能化签约服务新模式。

3.推进远程医疗进乡村。建设完善基层远程医疗服务网络，推进基层远程医疗门诊、健康随访、健康监测、会诊、查房等服务，推动优质医疗资源向乡村及偏远地区延伸。

　　（七）强化卫生健康统计调查分析应用体系
　　坚持依法统计、应用导向、质量优先、创新发展，健全卫生健康统计调查体系，强化信息化在提升统计数据质量、推进统计数据共享应用、发挥统计监督职能等方面的作用，为卫生健康事业高质量发展提供统计决策支撑。
　　持续完善统计调查体系。推动统计工作以治病为中心转向以健康为中心，统计领域从医疗卫生扩展至健康服务，逐步构建涵盖卫生健康资源、医疗健康服务、公共卫生安全、居民健康水平、健康影响因素、行业综合治理、健康产业发展等全人群全生命周期的卫生健康统计调查体系，建立贯穿预防、治疗、康复、健康管理等环节的居民健康统计信息闭环。依托全民健康信息平台及国家卫生健康统计网络直报系统，统筹相关数据资源，开展“一老一小”、健康预期寿命测算等常态化统计分析应用。依法制（修）订统计调查制度，规范实施国家卫生健康统计调查制度、国家中医药综合统计制度，建立健全动态调整的健康中国行动监测评估指标体系，推动落实健康中国行动监测评估和考核工作，加强生育和人口形势监测研判，加快建立健康服务业和健康产业核算机制，完善全国卫生服务统计调查及居民卫生服务监测、全民健康信息化统计调查与应用评价。
　　加强统计数据质量控制。认真贯彻落实《[统计法](https://www.pkulaw.com/chl/6e3bca4bbba3f66ebdfb.html?way=textSlc)》《[标准化法](https://www.pkulaw.com/chl/36bdf552f9bcf06cbdfb.html?way=textSlc)》等法律法规及配套规范要求，完善统计数据标准制度，规范统计数据形成机制，推进卫生健康统计工作数字化转型，推动统计数据采集方式转变，增加从业务系统中直接获取统计数据的比重，提高数据准确性、时效性。建立健全科学规范、权责明晰的卫生健康统计数据质量评估和反馈机制，运用新一代信息技术，创新统计数据质量控制方式，整合统计数据资源，加强分析应用，定期开展统计数据质量评估，推动统计数据全流程质量控制。
　　强化统计数据共享应用。依托全民健康信息平台，加快整合业务重叠、分散独立的信息系统，整合各相关部门统计调查数据，最大限度发挥统计数据分析价值。建立完善卫生健康统计信息发布机制，通过相关渠道向机构和社会公众开通查询功能，及时高效发布统计信息。制定统计信息共享管理服务规范，建立健全统计信息资源目录和共享机制，积极推动统计信息共享、交换和应用研究。推进统计数据安全有序开放，探索建立与高水平医疗卫生机构及高等院校、科研院所、企业等社会第三方智库的合作机制，围绕重点难点问题，开展统计调查大数据融合应用，发挥行为分析、态势感知、预测预报、政策评价与模拟等方面辅助决策作用。
　　全面提升统计监督效能。加快构建系统完整、协同高效、约束有力的卫生健康统计监督体系。督导各级卫生健康行政部门严格执行统计调查制度，充分发挥信息化在统计监督中的作用，依法依规开展统计调查。稳定统计调查队伍，增强卫生健康统计归口管理部门及统计技术支撑机构的专业力量，为依法开展统计调查工作提供基础条件保障。
　　（八）夯实网络与数据安全保障体系
　　坚持发展与安全并重，完善网络安全和数据安全制度，围绕网络与数据安全全链条、全要素、全周期加强教育培训和宣贯，加大网络安全投入，切实防范化解风险，提高安全防护能力，不断完善网络安全和数据安全综合防范体系。
　　全面落实网络安全和数据安全相关法规标准。贯彻落实《[网络安全法](https://www.pkulaw.com/chl/4dce14765f4265f1bdfb.html?way=textSlc)》《[数据安全法](https://www.pkulaw.com/chl/1dd3a2fb498ecb3ebdfb.html?way=textSlc)》《[个人信息保护法](https://www.pkulaw.com/chl/d653ed619d0961c0bdfb.html?way=textSlc)》《[密码法](https://www.pkulaw.com/chl/9aa7c80922b4582cbdfb.html?way=textSlc)》《[关键信息基础设施安全保护条例](https://www.pkulaw.com/chl/34a2a5e14df5feb9bdfb.html?way=textSlc)》及配套标准规范要求，履行好法律赋予的网络安全、数据安全监管权和行政执法权。在严格落实网络安全等级保护制度及商用密码应用等基础安全保障制度的基础上，以关键信息基础设施安全为重点，落实数据出境安全管理制度，加强医疗设备相关网络和数据安全监管，全面落实网络安全管理要求。研究制定卫生健康信息管理办法和相应的标准规范，对合理使用数据提供合规指南，对违规行为及时予以纠正。
　　完善网络安全和数据安全责任体系和管理制度。落实党委（党组）网络安全和数据安全责任制，压实主体责任，落实[网络安全审查办法](https://www.pkulaw.com/chl/2c77a9b321683224bdfb.html?way=textSlc)，强化绩效考核和评价机制。加强技术支撑机构建设，完善行业网络安全和数据安全监测、检查和通报机制，增强网络安全和数据安全应急响应能力，完善人防、物防、技防、制防、时防相关制度和措施，全面提升网络安全和数据安全管理能力。探索信息技术应用创新试点示范，提升供应链安全管理能力。
　　构建卫生健康行业网络可信体系。建设一批医疗卫生机构商用密码应用示范，全面推广商用密码应用，完善卫生健康行业商用密码应用体系。建设各类医疗卫生机构、人员和患者可信数字身份管理系统，实现医患可信身份电子认证和电子签名，保证访问、处理数据的用户身份真实，确保网络行为可管、可控、可溯源。完善卫生健康行业电子认证服务体系，实现电子认证服务跨区域互信互认。

专栏五 关键信息基础设施安全保护工程

1.建设关键信息基础设施监测预警和威胁分析平台。完善行业资产库及相关知识库，建设安全态势感知平台，结合威胁信息分析研判机制，支持威胁信息、漏洞信息与资产信息的快速匹配，提升监测预警与快速处置能力。

2.制定数据分类分级指南，确定核心数据、重要数据和一般数据目录，提出相应保护的管理要求和技术措施，提升数据安全和个人信息保护能力。

3.建立关键信息基础设施首席网络安全官、专职安全管理员、关键岗位人员分类培训体系，加强实战，持证上岗。

　　**四、**优先行动
　　遵循全民健康信息化发展规律，坚持整体推进与重点突破相结合，在不断夯实信息化基础设施建设，持续推进“互联网＋医疗健康”便民服务与健康医疗大数据应用发展的基础上，通过优先开展一批行动，着力在信息互通共享、健康中国建设、重点人群智能服务等方面取得突破性进展，推动全民健康信息化向数字健康跃升，增强人民群众获得感、幸福感和安全感。
　　（一）互通共享三年攻坚行动
　　以普及应用居民电子健康码为抓手，建立居民以身份证号码为主、其他证件号码为补充的唯一主索引，推动“一码通用”。依托区域全民健康信息平台，推动检查检验结果互通共享。基于省统筹区域全民健康信息平台，推进省级影像云存储基础设施建设，实现检查检验数据智能、高效、融合、经济的存储和传输。国家和省级建设电子病历、居民电子健康档案索引库，地市级及县级建成与区域全民健康信息平台相衔接的全量电子病历、居民电子健康档案信息库，依托国家全民健康信息平台，在保障网络安全和保护个人信息的前提下，推进电子病历、居民电子健康档案跨省查询。通过移动端应用，各省实现向本人提供电子病历、居民电子健康档案实时查询服务。
　　（二）健康中国建设（行动）支撑行动
　　健全健康中国行动统计调查制度，进一步构建全面覆盖健康中国建设、健康中国行动主要指标的健康中国监测评估指标体系。以数字赋能为抓手，推进健康中国建设（行动）监测评估、考核信息系统与全国爱国卫生资源管理系统的建设，逐步实现数据跨部门、跨层级共建共享，增强数据时效性。完善健康中国监测考核和决策分析体系，开展动态监测和定期评估，探索建立健康中国行动综合指数和分类指数，及时掌握健康中国建设（行动）实施进度和成效，提升健康中国行动实施效果的精准性和有效性。推动“互联网＋精准健康科普”，探索建立健康科普数据平台，满足全生命周期不同人群对不同健康科普的需求。鼓励各地依托现有资源，运用新媒体，推进全民健康生活方式行动，强化家庭和高危个体健康生活方式指导和干预。
　　（三）智慧医院建设示范行动
　　按照《全国医院信息化建设标准与规范》要求，加强医院信息化标准化规范化建设。基于医院信息平台整合医院内部信息系统，构建线上线下一体化服务，提升患者就医满意度。推进医疗数据统一管理，加快临床诊疗无纸化进程。鼓励应用临床诊疗辅助决策系统优化医疗服务场景。优化门急诊就医流程，推进院前与院内急救无缝衔接。支持各地探索建立统一的“互联网＋医疗健康”服务入口。推动提升中医医院智慧化水平，鼓励智慧中药房建设，提高中医药数字便民服务能力。鼓励医疗机构积极拓展智慧管理创新应用，提升医院运营管理效率，支持医疗、服务、管理、科研一体化监管。
　　（四）重点人群智能服务行动
　　以社区与家庭为基础，搭建医养康养信息化服务平台，积极构建医防融合体系，强化数字医养康养服务应用。依托全民健康信息平台，优化妇幼健康信息系统、人口统筹管理业务应用系统，建设全国托育服务信息管理系统，完善全国老龄健康信息管理系统，推进数据统一接入，实现业务协同、数据共享和统一监管，不断推进云上妇幼、智慧养老与智慧托育服务，建设可视化风险地图，强化重点人群和场所监测。完善全国一体化职业健康信息管理平台，推进职业健康信息化建设应用，实现职业健康信息的横向联通、上下联动和动态管理，提高职业病危害风险监测预警与智能决策的支撑能力。
　　（五）药品供应保障智慧监测应对行动
　　实施国家药品供应保障与使用评价能力提升工程，健全国家药品供应保障综合管理平台功能，完善药品使用监测、临床综合评价和短缺药品预警信息化支撑体系。建设国家和省两级药品使用监测系统，构建国家、省、市、县四级药品使用监测网络，加强医疗机构药品智能化监测及数据采集能力，推动实现全国二三级公立医院全覆盖和80％基层公立医疗卫生机构基本覆盖的总体目标。优化短缺药品多源信息采集和供应业务协同应用系统建设，有效提升监测预警、分析研判、应对处置的综合能力，不断健全短缺药品保供稳价长效机制。以基本药物、急（抢）救药和儿童、老年人、罕见病患者等重点人群用药为重点，健全药品供求大数据监测系统，不断健全药品供应保障制度体系。建设药物政策辅助决策系统，实现药品应用编码（YPID）标准的全面推广应用和多码联动，提升药政治理数字化水平。
　　（六）数字公卫能力提升行动
　　统筹推进与相关部门信息系统联通，提高监测预警、实时分析、集中研判和辅助决策的能力。建立集中统一高效的应急指挥辅助决策体系，提升疫情应急处置能力和精准防控水平。加强公共卫生信息系统与各级全民健康信息平台及各级医院、基层医疗卫生机构等业务信息系统的高效对接与数据共享，完善传染病疫情和突发公共卫生事件监测相关标准，提高医防信息融合水平。运用数字技术建设“智慧食安”，实现风险动态可视化，支撑风险预警、趋势研判和科学决策，全面提升食品安全监管和服务能力。依据全国食品污染和食源性疾病时空分布及变化规律，绘制主要污染物和食源性疾病风险地图，强化监测和风险预警能力，加强食品安全信息惠民服务。加强健康码标准规范使用，强化赋码和转码规则规范实施，推进互通互认、一码通行。以数字化转型打造“数智卫监”，实现风险可预警、数据可分析、监管可联动，提升事中事后监管规范化、精准化和智能化水平。
　　（七）“互联网＋中医药健康服务”行动
　　统筹建设国家和省级中医药数据中心，加强全民健康保障信息化工程中医药业务平台应用与完善，强化与全民健康信息平台互联互通。优化升级中医馆健康信息平台，扩大联通范围，推进与基层医疗卫生机构信息系统集成应用。深化数字中医药体系。鼓励地方加强中医医院信息化建设，加快信息基础设施提档升级，推动构建以中医电子病历、电子处方等为重点的基础数据库，推动一体化共享、一站式结算等数字化便民服务，鼓励医疗机构研发应用名老中医传承、智能辅助诊疗系统等具有中医药特色的信息系统。
　　（八）数据安全能力提升行动
　　落实数据安全法规制度和标准，严格核心数据管控，加强重要数据保护，规范一般数据管理。加强重要数据和个人信息出境安全评估、监测和检查，及时发现安全隐患，防止数据违规出境。建设数据安全态势感知平台，丰富技术检查监测手段，组建行业专门技术支撑机构，落实风险评估、监测预警和应急处置等制度，提升网络安全和数据安全保护能力。开展行业网络安全比武竞赛、攻防演练，提升行业实战对抗能力，加强行业网络安全人才培养。推动分布式存储、多方安全计算等关键技术研发和应用，运用人工智能、区块链等新一代信息技术进行数据安全防护。做好个人信息安全保护，重点保护大规模个人信息和敏感个人信息。加强知识普及和法规宣贯，组织教育培训，提高全行业人员网络安全和个人信息保护的意识和能力。

　　**五、**组织实施
　　（一）加强组织领导，强化统筹协调
　　坚持党对网络安全和信息化工作的集中统一领导，把信息化发展摆到工作全局更加突出的位置，发挥网络安全和信息化工作领导小组决策和统筹协调作用，坚持“一把手”亲自抓、负总责，将其纳入重点工作计划和列入各部门年度考核指标，形成“一盘棋”工作格局，构建数据资源一体化统筹管理体系，协调推进各项重大任务、重点工程和优先行动。
　　（二）完善规章制度，健全政策体系
　　统筹全民健康信息化制度建设，制定与发展相匹配的医学伦理、数据确权、数据交易、网络安全等规章制度，健全全民健康信息化建设发展的政策体系，完善适应卫生健康信息化行业特点的技术创新、知识产权、数据共享、安全保障等标准规范。强化执法监督与能力建设。各地要健全投入保障机制，切实推动全民健康信息化建设可持续发展。
　　（三）加强队伍建设，强化人才支撑
　　注重拓宽人才培养渠道，充分发挥高等院校、科研院所特别是国家健康医疗大数据研究院等机构在全民健康信息化工作中的智力支撑作用，加快建立适应行业特点的新一代信息技术创新应用人才队伍培养体系。研究制定卫生健康信息化人员配备标准，突出加强数据分析、网络安全等技术人员配备。完善人才使用培训机制，针对不同层次信息化人员的岗位需求制定系列培训计划。完善专家决策咨询机制，发挥专家在前瞻性研究、信息化规划、重大项目论证、新技术应用等领域的智力支撑。鼓励各地探索建立首席信息官制度。加强对生物医学工程、医学信息技术等新专业和交叉型人才建设，营造促进实用型人才与复合型人才协调发展的政策环境，为推动全民健康信息化高质量发展提供有力支撑。
　　（四）严格监督评估，强化任务落实
　　支持将医院信息化互联互通情况纳入医院绩效考核、医院等级评审等工作中，将全民健康信息化建设发展情况纳入卫生健康部门的考评范围，与经费拨付、设备配置、绩效评价和人员考核相结合。要加强调查研究，督促指导信息化工程与重点任务的实施，分析研判风险，及时解决重点领域与关键环节存在的问题，适时发布年度发展报告，切实抓好规划落地落实。
　　（五）深化国际交流，实现共赢发展
　　坚持安全发展、协同共进的原则，参与全球数字健康国际合作，加强与多双边平台机制的对接，深化与国际组织、产业联盟和科研机构的交流合作，推广数字健康相关技术、产品、标准、服务、规则和共识，注重对国际卫生健康信息化应用标准的跟踪、评估和转化，推动我国自主技术与全球同步发展，探索国际健康医疗发展合作新模式，不断提升我国全民健康信息化应用水平、产业核心竞争力和国际影响力。

#### 背景资料

##### 法规解读

[《“十四五”全民健康信息化规划》解读](https://www.pkulaw.com/lawexplanation/304829c1b16a61a7148dc3a596ee6489bdfb.html?way=textRightFblx)

#### 本篇引用的法规

##### 中央法规

[网络安全审查办法(2021)](https://www.pkulaw.com/chl/2c77a9b321683224bdfb.html?way=textRightFblx)

[中华人民共和国个人信息保护法](https://www.pkulaw.com/chl/d653ed619d0961c0bdfb.html?way=textRightFblx)

[关键信息基础设施安全保护条例](https://www.pkulaw.com/chl/34a2a5e14df5feb9bdfb.html?way=textRightFblx)

[中华人民共和国数据安全法](https://www.pkulaw.com/chl/1dd3a2fb498ecb3ebdfb.html?way=textRightFblx)

[中华人民共和国国民经济和社会发展第十四个五年规划和2035年远景目标纲要](https://www.pkulaw.com/chl/dad03bd96074290cbdfb.html?way=textRightFblx)

[国家卫生健康委办公厅、国家中医药局办公室关于加强全民健康信息标准化体系建设的意见](https://www.pkulaw.com/chl/6c5528153b4fe48cbdfb.html?way=textRightFblx)

[中华人民共和国密码法](https://www.pkulaw.com/chl/9aa7c80922b4582cbdfb.html?way=textRightFblx)

[国务院办公厅关于促进“互联网+医疗健康”发展的意见](https://www.pkulaw.com/chl/37395b41f6f018e4bdfb.html?way=textRightFblx)

[更多](https://www.pkulaw.com/AssociationMore/chl/b893d7cf8363a392bdfb/2_100.html?way=textRightFblx)

©北大法宝：（[www.pkulaw.com](https://www.pkulaw.com/)）专业提供法律信息、法学知识和法律软件领域各类解决方案。北大法宝为您提供丰富的参考资料，正式引用法规条文时请与标准文本核对。 欢迎查看所有[产品和服务](http://www.pkulaw.net/)。
[法宝快讯： 如何快速找到您需要的检索结果？ 法宝 V6 有何新特色？](http://www.pkulaw.com/helps/69.html)

扫描二维码阅读原文

原文链接：<https://www.pkulaw.com/chl/b893d7cf8363a392bdfb.html>
